# Twelve-month outcomes of subretinal tenecteplase-assisted vitrectomy for large submacular hemorrhage secondary to polypoidal choroidal vasculopathy: a retrospective consecutive case series

**DOI:** 10.3389/fmed.2026.1981363

**Published:** 2026-10-20

**Authors:** Ling Ai, Xindan Meng, Shan He, Shiman Yuan, Yixia Zhang, Yi Wang

**Affiliations:** 1 Jinan University, Guangzhou, China; 2 Chongqing Nanping Aier Eye Hospital, Chongqing, China; 3 Henan Medical University, Xinxiang, China; 4 Chongqing Aier Eye Hospital, Chongqing, China

**Keywords:** anti-vascular endothelial growth factor, pars plana vitrectomy, polypoidal choroidal vasculopathy, submacular hemorrhage, subretinal injection, tenecteplase, tissue plasminogen activator

## Abstract

**Background:**

To evaluate the safety, anatomical and visual outcomes, and prognostic factors of pars plana vitrectomy (PPV) with subretinal tenecteplase (TNK-tPA) injection and gas tamponade for submacular hemorrhage (SMH) secondary to polypoidal choroidal vasculopathy (PCV).

**Methods:**

In this retrospective case series, 10 eyes of 10 consecutive patients with PCV and fovea-involving SMH (>5 disc areas) underwent 25-gauge PPV, subretinal TNK-tPA injection (50 μg/0.05 mL at a single site in 7 eyes; multi-site injection totaling up to 100 μg in 3 eyes), and tamponade with 0.4 mL of pure C₃F₈ gas, followed by pro re nata anti-vascular endothelial growth factor (anti-VEGF) therapy. Best-corrected visual acuity (BCVA), central macular thickness (CMT), pigment epithelial detachment (PED), anti-VEGF treatment burden, and complications were evaluated over 12 months.

**Results:**

Complete subfoveal hemorrhage displacement was achieved in all eyes (100%) by postoperative day 1. At 12 months, median BCVA improved from 1.25 (IQR, 1.10–1.70) to 0.30 (IQR, 0.23–0.95) logMAR (*Z* = −2.617, *P* = 0.010), and mean CMT decreased from 1,006.0 ± 183.0 μm to 205.9 ± 48.2 μm (*t* = 12.44, *P* < 0.001). PED resolved in 90.0% of eligible eyes by month 2, with a mean of 1.8 anti-VEGF injections. The 12-month BCVA correlated with time to surgery (*r* = 0.84, *P* = 0.002) and hemorrhage area (*r* = 0.87, *P* = 0.001); given the strong collinearity between these variables (*r* = 0.918) and the small sample size, these associations are exploratory and hypothesis-generating. No TNK-tPA–related adverse events were observed.

**Conclusions:**

PPV with subretinal TNK-tPA injection and gas tamponade was associated with rapid hemorrhage displacement, visual improvement, and an observed mean of 1.8 anti-VEGF injections over 12 months in this small PCV cohort. Better visual outcomes were associated with shorter time to surgery and smaller hemorrhage area; these associations are exploratory given the observational design and the strong collinearity between these two factors. Larger controlled studies are needed to confirm safety and comparative effectiveness.

## 1 Introduction

Polypoidal choroidal vasculopathy (PCV), first described by Yannuzzi and colleagues (1), is traditionally classified as a distinct subtype of neovascular age-related macular degeneration (nAMD) and is increasingly recognized within the pachychoroid disease spectrum (2, 3). Characterized by branching vascular networks with terminal polypoidal dilations arising from the inner choroid (2, 4), PCV differs from typical nAMD in epidemiology, natural history, and treatment response (3), and accounts for 22%–55% of presumed nAMD in Asian populations (2–5).

Large SMH is a well-recognized complication of PCV because the polypoidal lesions are prone to rupture and profuse bleeding (2, 4), and it carries a particularly poor prognosis: untreated subfoveal hemorrhage leads to severe and largely irreversible visual loss (6).

The visual toxicity of SMH is multifactorial and time-dependent. Experimental studies have shown that subretinal blood damages photoreceptors within hours to days through a diffusion barrier effect, iron-related toxicity, and mechanical shearing from clot contraction, with irreversible outer retinal destruction documented as early as 24 h in animal models (7, 8). Fibrin contraction in particular exerts direct traction on the outer retina (8). Consistent with these mechanisms, the natural history of subfoveal SMH in nAMD is one of progressive fibrosis, disciform scarring, and profound vision loss (6, 9), providing a strong rationale for urgent hemorrhage removal.

Current evacuation strategies include pneumatic displacement with intravitreal gas, alone (10) or combined with intravitreal tissue plasminogen activator (tPA) (11), and pars plana vitrectomy with subretinal tPA injection, with or without perfluorocarbon liquid (12, 13) or concomitant anti-VEGF agents (14). For PCV-related SMH specifically, favorable anatomical and visual outcomes have been reported with vitrectomy plus subretinal recombinant tPA (alteplase) (15, 16) and with intravitreal tPA combined with ranibizumab and gas (17).

Tenecteplase (TNK-tPA) is a genetically engineered variant of alteplase with greater fibrin specificity, a longer plasma half-life, and greater resistance to plasminogen activator inhibitor-1 (18, 19). Intravitreal (20) and subretinal (21) TNK-tPA have shown encouraging results for SMH secondary to nAMD, and a recent prospective study reported the feasibility of vitrectomy with subretinal TNK-tPA and anti-VEGF therapy (22). However, clinical evidence on subretinal TNK-tPA for large PCV-related SMH remains limited. Although comparative studies have reported broadly comparable visual outcomes between subretinal and intravitreal delivery, their interpretation is limited by small sample sizes, baseline imbalances, and differences in study design (23, 24). Moreover, data on long-term anatomical and visual outcomes, safety, subsequent anti-VEGF treatment burden, and factors associated with visual prognosis after subretinal TNK-tPA treatment remain scarce. We therefore evaluated these outcomes during at least 12 months of follow-up after vitrectomy with subretinal TNK-tPA injection and gas displacement in patients with PCV and large SMH.

## 2 Materials and methods

### 2.1 Study design and patients

This retrospective interventional case series included consecutive patients with PCV and SMH treated at Chongqing Nanping Aier Eye Hospital between April 2024 and July 2025. The study adhered to the Declaration of Helsinki and was approved by the Institutional Review Board of Chongqing Nanping Aier Eye Hospital.

PCV was diagnosed by the presence of polypoidal lesions, with or without a branching vascular network, on indocyanine green angiography, in accordance with established criteria (3), with supportive optical coherence tomography (OCT) findings. The inclusion criteria were (1) fovea-involving SMH exceeding 5 disc areas; (2) active PCV, either treatment-naïve or previously treated; and (3) a minimum follow-up of 12 months. The exclusion criteria were SMH due to causes other than PCV (e.g., retinal arterial macroaneurysm, trauma, or myopic choroidal neovascularization) and previous vitreoretinal surgery. The patient screening and enrollment process is summarized in Figure 1.

**Figure 1:**
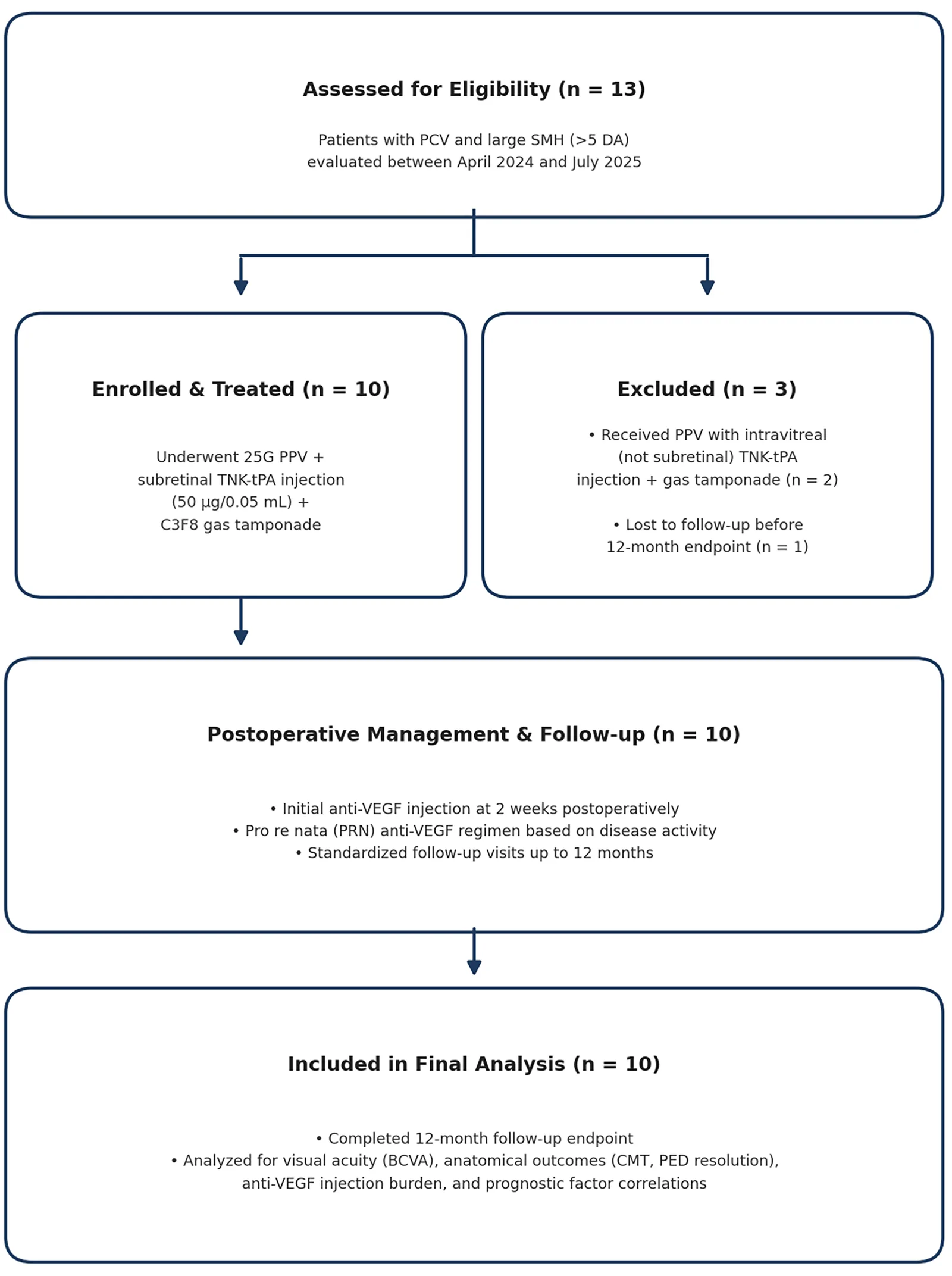
Patient screening and enrollment flow diagram. During the study period, 13 patients with PCV-related SMH were assessed at our center; 2 received PPV combined with intravitreal TNK-tPA injection and gas tamponade and were not included in this subretinal injection series; 11 underwent pars plana vitrectomy with subretinal TNK-tPA, of whom 1 was lost to follow-up before 12 months; the remaining 10 consecutive eligible eyes completed the prespecified 12-month outcome assessment and were analyzed. PCV, polypoidal choroidal vasculopathy; SMH, submacular hemorrhage; TNK-tPA, tenecteplase.

### 2.2 Surgical procedure

All surgeries were performed by an experienced vitreoretinal surgeon using standard three-port 25-gauge pars plana vitrectomy. Tenecteplase (TNK-tPA, 50 μg/0.05 mL per injection site) was injected into the subretinal space via a 41-gauge cannula, with the injection site selected at the area of thickest hemorrhage and deliberately avoiding the foveal center. Injection pressure and flow were controlled using the viscous-fluid (silicone oil) injection mode of the vitrectomy system, so that a controlled subretinal fluid wave was created to lavage and liquefy the clot under direct visualization. The total dose did not exceed 50 μg (0.05 mL) for single-site injection; when multiple sites were required for extensive hemorrhages, the total dose did not exceed 100 μg (0.1 mL), and the volume delivered within 1 disc diameter of the foveal center was limited to 0.05 mL. In this cohort, 7 eyes received a single-site injection of 50 μg (0.05 mL), and 3 eyes with extensive hemorrhage received multi-site injections (two sites in 2 eyes and three sites in 1 eye), with the total dose divided across sites and not exceeding 100 μg (0.1 mL). A fluid–air exchange was then performed, followed by the injection of 0.4 mL of pure (100%) C₃F₈ gas into the vitreous cavity. No active subretinal drainage was performed; the liquefied hemorrhage was displaced away from the fovea by gas tamponade alone. Postoperatively, patients remained in a supine position for 15 min on the operating table before starting face-down positioning (see Section 2.3).

### 2.3 Postoperative management

Postoperatively, patients maintained strict face-down positioning for 2 weeks (≥16 h/day) to displace the liquefied hemorrhage from the fovea. Intravitreal ranibizumab (0.5 mg/0.05 mL) was initiated 2 weeks postoperatively under a pro re nata (PRN) protocol. This 2-week interval was rationale-driven: (1) local health insurance reimbursement regulations and financial constraints in China; (2) progressive intraocular gas absorption by week 2, enhancing fundus visibility and OCT image quality; and (3) clinical evidence indicating that PCV lesion activity is characteristically diminished in the early phase following massive subretinal hemorrhage due to major polyp rupture, rendering the short-term risk of early reactivation or recurrent bleeding within the first 2 weeks low (25). Retreatment criteria were persistent or recurrent intraretinal or subretinal fluid, recurrent hemorrhage, or a vision-threatening pigment epithelial detachment (PED) involving the foveal center. Follow-up visits were scheduled at 1 day, 1 week, 2 weeks, and 1, 2, 3, 6, and 12 months after surgery; the 12-month visit was prespecified as the study endpoint. Patients were instructed to return promptly for any visual deterioration or ocular discomfort between scheduled visits.

### 2.4 Outcome measures

The outcomes were (1) displacement and absorption of SMH; (2) BCVA, measured with a standard decimal visual acuity chart and converted to logMAR units for analysis—counting fingers (CF) acuity was converted to 2.0 logMAR (equivalent to Snellen 20/2000), consistent with published quantifications of counting-fingers acuity (26, 27); (3) anatomical outcomes, including CMT, outer retinal integrity [external limiting membrane (ELM) and ellipsoid zone (EZ)], and PED resolution on spectral-domain OCT; (4) treatment burden, defined as the total number of anti-VEGF injections administered over 12 months; and (5) safety, assessed from intraoperative and postoperative complications. Using ImageJ software (National Institutes of Health, Bethesda, MD, USA), the boundaries of both the hemorrhage and the optic disc were manually delineated on SLO fundus images. The SMH area was quantified in disc areas (DA) by calculating the ratio of the measured hemorrhage area to the optic disc area. Spectral-domain optical coherence tomography (SD-OCT) imaging was performed using the Cirrus HD-OCT system (software version 9.5; Carl Zeiss Meditec, Inc., Dublin, CA, USA), and retinal structural parameters were acquired using the standardized Macular Cube 512 × 128 scan protocol, which covers a 6 mm × 6 mm area centered on the fovea. Complete PED resolution was defined on OCT as complete flattening of the RPE line with no residual pigment epithelial detachment. Outer-retinal integrity was assessed on these volume scans, and the horizontal B-scan through the foveal center was used for grading. ELM and EZ integrity were evaluated jointly: disruption was defined as the segment of the scan in which both the ELM and the EZ lines were absent, measured with the built-in caliper tool of the OCT device software and expressed as a proportion of the 6 mm scan length, at baseline and at the 12-month visit. Of the 2 eyes with concurrent vitreous hemorrhage, the hemorrhage was mild in 1 eye and did not preclude macular visualization; in the other eye, baseline outer-retinal status was graded from the most recent macular OCT obtained 5 days before the vitreous hemorrhage developed. Submacular scar was defined as a subretinal hyperreflective nodule within a 2.0 mm radius centered on the fovea. Image grading was conducted independently by two experienced retina specialists masked to clinical parameters (baseline and follow-up BCVA, time to surgery) and each other's readings, with disagreements resolved by consensus.

### 2.5 Statistical analysis

Statistical analyses were performed using SPSS Statistics, version 26.0 (IBM Corp.). Continuous variables are presented as mean ± standard deviation or median (interquartile range), as appropriate. Hemorrhage area was measured in disc areas (DA), and time to surgery was defined as the interval from symptom onset to surgery. Preoperative and postoperative logMAR BCVA values were compared with the Wilcoxon signed-rank test. For CMT, the paired *t* test was used after confirming approximate normality of the paired differences (Shapiro–Wilk *W* = 0.918, *P* = 0.34); a Wilcoxon signed-rank test yielded a concordant result (*P* = 0.002). Spearman correlation and univariable linear regression were used to assess associations between final logMAR BCVA and candidate predictors; sex was coded as a binary indicator (male = 0, female = 1) for correlation analyses. Given the small sample, multivariable analysis was limited to exploratory two-covariate models (baseline logMAR BCVA and time to surgery; hemorrhage area and time to surgery). Residuals and variance inflation factors were assessed. No adjustment for multiple comparisons was applied given the exploratory nature of these analyses, and adjusted estimates are reported descriptively with 95% confidence intervals. A two-sided *P* < 0.05 was considered statistically significant. Effect estimates for paired changes are reported with 95% confidence intervals (mean change for CMT; Hodges–Lehmann median change with a bootstrap percentile confidence interval for logMAR BCVA). Proportions are reported with exact Clopper–Pearson 95% confidence intervals. For the principal univariable associations, influence diagnostics (leverage and Cook's distance) and leave-one-out sensitivity analyses were performed.

## 3 Results

### 3.1 Baseline characteristics

During the study period, 13 patients with PCV-related SMH were assessed at our center: 2 received PPV combined with intravitreal TNK-tPA injection and gas tamponade and were not included in this subretinal injection series, and 11 underwent PPV with subretinal TNK-tPA, of whom 1 was lost to follow-up before 12 months. The remaining 10 consecutive eligible eyes completed the prespecified 12-month assessment and constituted the analysis cohort (Figure 1).

A total of 10 eyes from 10 consecutive patients (6 males and 4 females) were included in this study. The mean age of the patients was 65.0 ± 11.7 years (range, 49–87 years). At baseline, the mean preoperative submacular hemorrhage (SMH) area was 30.8 ± 22.7 disc areas (DA) (range, 5.03–71.92 DA), and the mean preoperative CMT was 1,006.0 ± 183.0 µm. The median preoperative BCVA was 1.25 (IQR, 1.10–1.70) logMAR. The median time from symptom onset to surgery was 7.00 days (IQR, 5.25–11.00; range, 2–60 days). Baseline lens status was pseudophakic in 3 eyes, clear in 2 eyes, and showed pre-existing lens opacity in 5 eyes. Two eyes had been previously treated with anti-VEGF injections (1 and 2 injections; conbercept in one eye and ranibizumab in one eye); the remaining 8 eyes were treatment-naïve. All 10 eyes had hemorrhagic PED at baseline, 2 eyes (20%) had concurrent vitreous hemorrhage, and the hemorrhage extended beyond the macular arcade in 8 eyes (80%). Baseline ELM/EZ disruption involved a median of 96% (range, 60%–100%) of the macular scan. Baseline demographic and clinical characteristics are summarized in Table 1.

**Table 1:** Baseline characteristics.

Parameter	Value/Metric
Total Eyes (*N*)	10
Age (years)	65.00 ± 11.72 (49–87)
Gender (Male/Female)	6/4
Laterality (Right/Left), *n*	6/4
Lens status (pseudophakic/clear/lens opacity), *n*	3/2/5
Treatment-naïve/previously treated, *n*	8/2
Prior anti-VEGF injections in treated eyes, *n*	1 and 2 (conbercept in 1 eye, ranibizumab in 1 eye)
Preoperative SMH Area (DA)	30.82 ± 22.71 (5.03–71.92)
Hemorrhage extending beyond the macula, *n* (%)	8 (80)
Vitreous hemorrhage, *n* (%)	2 (20)
Baseline PED, hemorrhagic, *n* (%)	10 (100)
Baseline ELM/EZ disruption, median % (range)	96 (60–100)
Preoperative LogMAR BCVA	1.25 (IQR, 1.10–1.70)
Preoperative CMT (μm)	1,006.00 ± 182.99
Time to surgery (days)	7.00 (5.25–11.00) (2–60)
Follow-up duration (months)	15.50 ± 2.99

Data are presented as mean ± SD or median (IQR), as appropriate. DA, disc area; BCVA, best-corrected visual acuity; CMT, central macular thickness; PED, pigment epithelial detachment; ELM, external limiting membrane; EZ, ellipsoid zone; IQR, interquartile range; SD, standard deviation. ELM/EZ disruption was quantified as the proportion of the macular scan length with loss of the ELM/EZ line on spectral-domain OCT.

### 3.2 Visual outcomes

Preoperative decimal BCVA ranged from counting fingers at 20 cm to 0.1 and improved to 0.02–1.0 at 12 months. Median logMAR BCVA improved significantly from 1.25 (IQR, 1.10–1.70) to 0.30 (IQR, 0.23–0.95) (*Z* = −2.617, *P* = 0.010) (median improvement, 0.80 logMAR; Hodges–Lehmann estimate with bootstrap 95% CI, 0.40–1.20) (Table 2). The 12-month logMAR BCVA was strongly correlated with time to surgery (Spearman *r* = 0.84, *P* = 0.002) (Table 3), with a shorter interval associated with better visual acuity.

**Table 2:** Visual and anatomical outcomes summary.

Outcome metric	Preoperative	12-month follow-up	Test statistic	*P* value
LogMAR BCVA	1.25 (IQR: 1.10–1.70)	0.30 (IQR: 0.23–0.95)	*Z* = −2.617	0.010
Mean CMT (μm)	1,006.0 ± 183.0	205.9 ± 48.2	*t* = 12.44	<0.001

BCVA, best-corrected visual acuity; CMT, central macular thickness; IQR, interquartile range. LogMAR BCVA was compared using the Wilcoxon signed-rank test, and CMT using the paired *t* test after confirmation of approximate normality of the paired differences (Shapiro–Wilk *P* = 0.34); a Wilcoxon signed-rank test for CMT yielded a concordant result (*P* = 0.002).

**Table 3:** Factors associated with 12-month visual outcomes.

Variable	*r*	*P*	Univariable *β* (95% CI)	*P*	Model A β (95% CI)	*P*	Model B β (95% CI)	*P*
Baseline logMAR BCVA	0.37	0.294	0.394 (−0.398 to 1.186)	0.284	0.200 (−0.365 to 0.766)	0.430	—	—
Female sex	0.58	0.082	0.600 (−0.134 to 1.334)	0.096	—	—	—	—
Age (years)	−0.29	0.418	−0.015 (−0.052 to 0.022)	0.388	—	—	—	—
Hemorrhage area (DA)	0.87	0.001	0.020 (0.009 to 0.032)	0.004	—	—	0.013 (0.001 to 0.025)	0.038
Preoperative CMT (μm)	0.67	0.035	0.0015 (−0.0007 to 0.0037)	0.155	—	—	—	—
Time to surgery (days)	0.84	0.002	0.026 (0.009 to 0.042)	0.007	0.024 (0.007 to 0.042)	0.014	0.015 (−0.001 to 0.031)	0.061

Model A included time to surgery and baseline logMAR BCVA; Model B included time to surgery and hemorrhage area. Multivariable analyses were exploratory because of the small sample size. BCVA, best-corrected visual acuity; DA, disc area; CMT, central macular thickness; CI, confidence interval. No adjustment for multiple comparisons was applied given the exploratory nature of the analyses; adjusted estimates are descriptive and hypothesis-generating, particularly given the strong collinearity between hemorrhage area and time to surgery (Spearman *r* = 0.918).

### 3.3 Anatomical and microstructural outcomes

By postoperative day 1, complete displacement of the subfoveal clot was observed in all 10 eyes (100%; exact 95% CI, 69.2%–100%) after subretinal thrombolysis and gas tamponade. No submacular scar formation was observed during follow-up. Baseline ELM/EZ disruption involved a median of 96% (range, 60%–100%) of the macular scan. At 12 months, the ELM/EZ was restored to intact in 2 eyes (20%), while the remaining 8 eyes showed residual disruption of 21%–100% (median 57%). Residual disruption was most severe in the 3 eyes treated more than 10 days after symptom onset (76%–100%), whereas eyes treated within 10 days showed less disruption (median 31%), including the 2 eyes with complete restoration. Baseline hemorrhagic PED (present in all 10 eyes) resolved completely in 9 eyes (90.0%; exact 95% CI, 55.5%–99.7%) by postoperative month 2. Mean CMT decreased from 1,006.0 ± 183.0 μm preoperatively to 205.9 ± 48.2 μm at the 12-month visit (paired t test, *t* = 12.44, *P* < 0.001; Wilcoxon signed-rank *P* = 0.002); mean decrease, 800.1 μm (95% CI, 654.6–945.6) (Table 2). Representative multimodal images are shown in Figures 2, 3. A representative case with a less favorable visual outcome (BCVA of 1.2 logMAR at 12 months after surgery performed 15 days after symptom onset, persistent complete ELM/EZ disruption) is shown in Figure 4.

**Figure 2:**
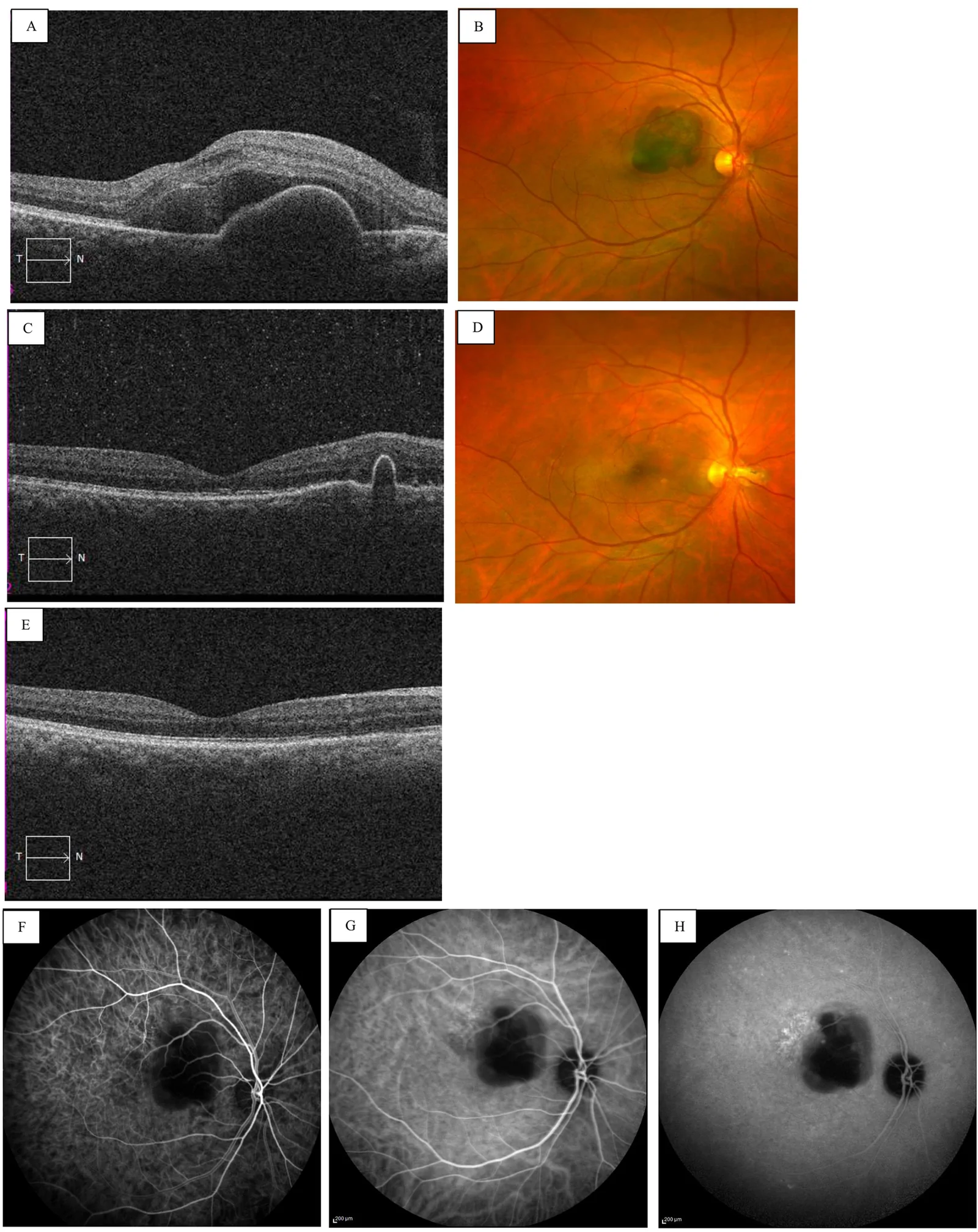
Multimodal imaging of a 52-year-old man with PCV-related SMH treated 2 days after symptom onset. Decimal BCVA improved to 1.0 at the 12-month follow-up. A, B: Preoperative evaluation. **(A)** OCT reveals macular subretinal hemorrhage/fluid with pigment epithelial detachment (PED) and ellipsoid zone disruption. **(B)** SLO image shows a large macular dark-red subretinal hemorrhage extending superiorly and nasal to the optic disc. **(C,D)**: Postoperative 2-week follow-up. **(C)** OCT shows substantial hemorrhage absorption and foveal contour restoration with a residual focal RPE elevation. **(D)** SLO image confirms marked hemorrhage resolution with yellowish absorptive changes. **(E)**: Postoperative 1-year OCT demonstrating complete lesion resolution, intact foveal architecture, and a restored ellipsoid zone, accompanied by mild outer retinal atrophic changes. F-H. Preoperative indocyanine green angiography (ICGA). **(F)** Early-phase (0:20) revealing the abnormal choroidal vascular network and early polypoidal hyperfluorescence. **(G)** Mid-phase (2:12) clearly highlighting the hyperfluorescent polypoidal lesions and branching vascular network (BVN) in the macular region. **(H)** Late-phase (12:16) showing characteristic late leakage from the polypoidal lesions with decreased surrounding choroidal fluorescence.

**Figure 3:**
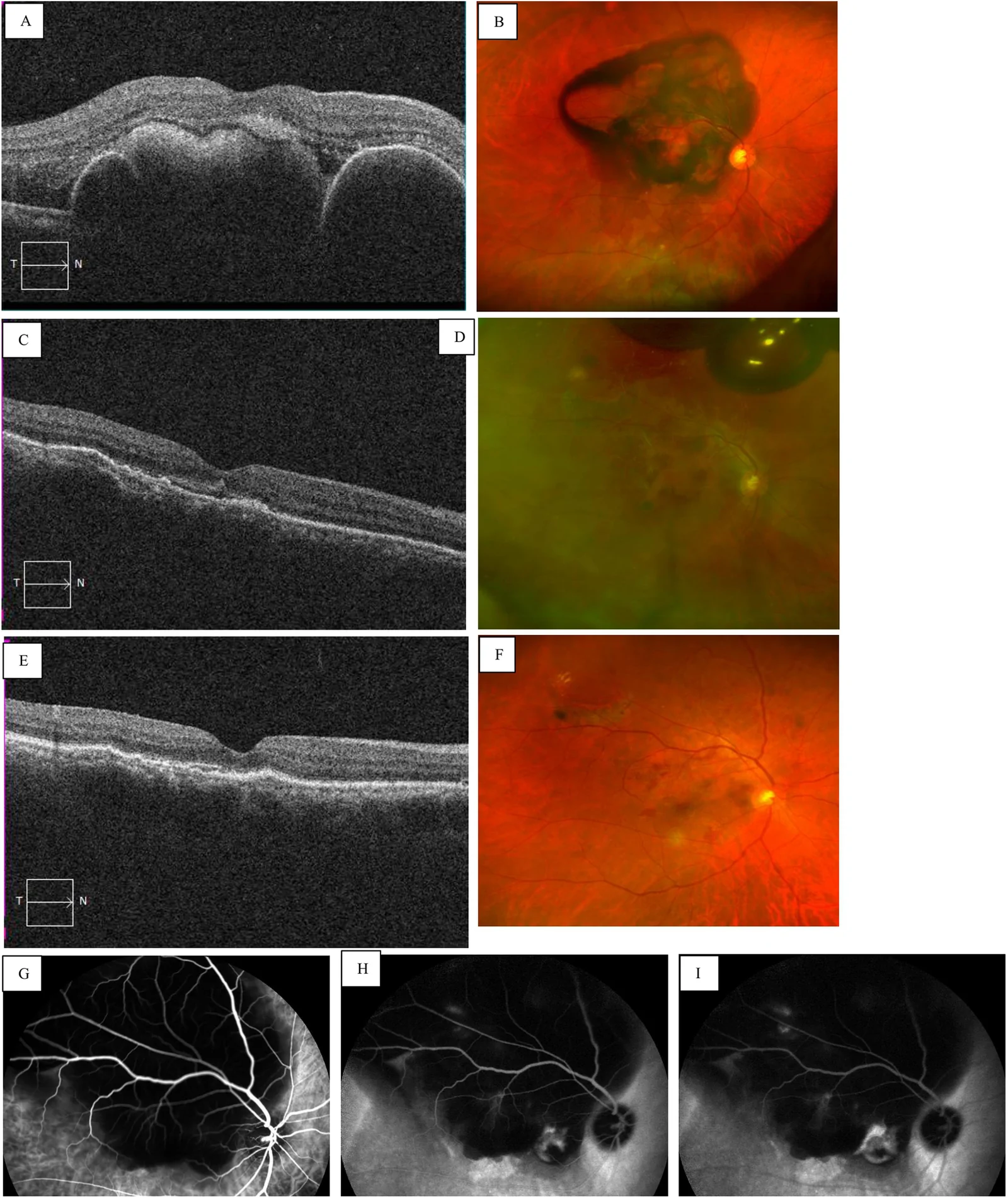
Multimodal imaging of a 63-year-old man with PCV-related SMH treated 1 week after symptom onset. Decimal BCVA improved to 0.6 at the 12-month follow-up. **(A,B)**: Preoperative evaluation. **(A)** OCT displays a prominent macular pigment epithelial detachment (PED), hyperreflective subretinal hemorrhage/fluid, and ellipsoid zone disruption. **(B)** SLO shows a large dark-red macular subretinal hemorrhage extending superiorly and nasal to the disc. **(C,D)**: Postoperative 2-week follow-up. **(C)** OCT demonstrates marked hemorrhage clearance, retinal reattachment, and residual low-lying RPE elevation. **(D)** SLO confirms substantial hemorrhage absorption with superior gas bubbles and clearing macular background. **(E,F)**: Postoperative 1-year follow-up. **(E)** OCT reveals complete lesion resolution, restored foveal contour, and intact RPE and ellipsoid zone layers. **(F)** SLO shows complete hemorrhage resolution with subtle macular RPE pigmentary changes. G–I: Preoperative dynamic ICGA sequence. **(G)** Early phase (0:37s): Shows central hemorrhage-induced blocked fluorescence with peripheral dilated choroidal vessels and branching vascular network (BVN). **(H)** Late phase (14:57s): Delineates a distinct ring-like hyperfluorescent polyp inferior-nasal to the disc with “central washout.” **(I)** Delayed phase (19:54s): Exhibits persistent ring-like polyp staining and late fluorescence. The subretinal TNK-tPA injection procedure is shown in Supplementary Video S1.

**Figure 4:**
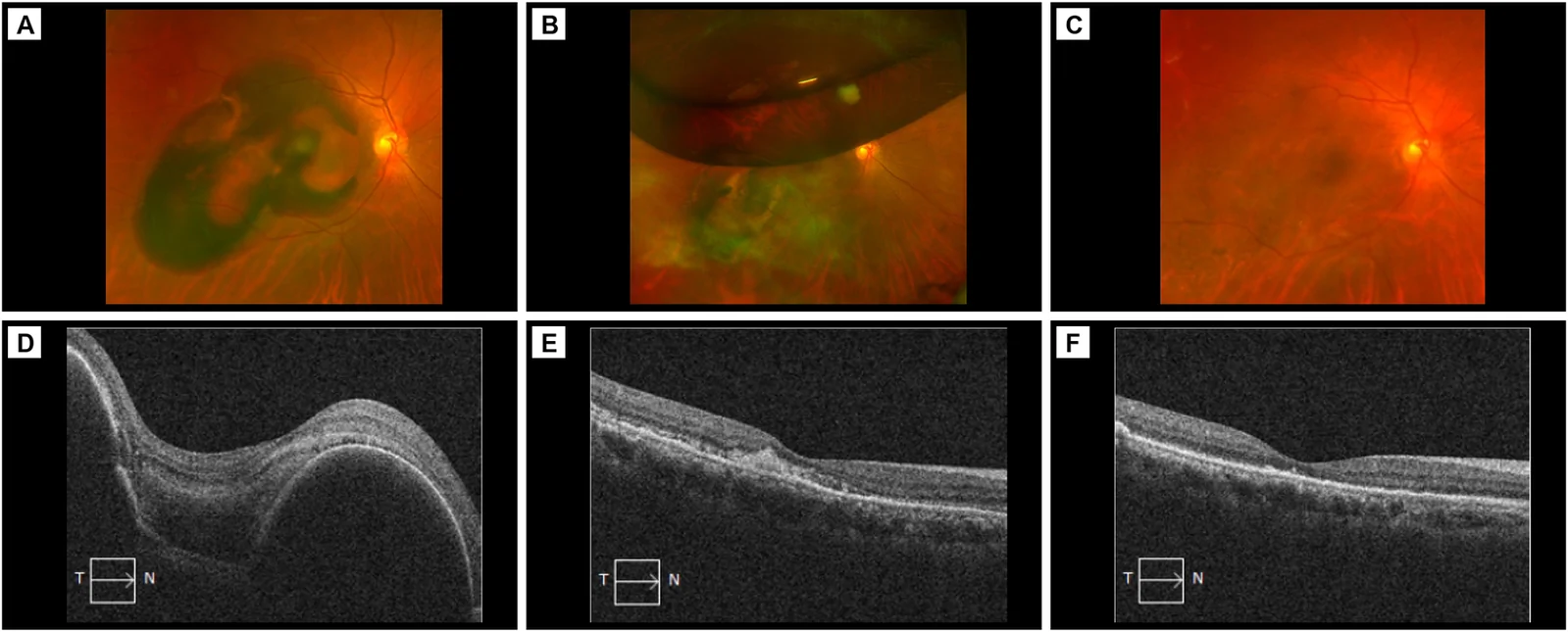
Representative case with a poorer visual outcome (a 64-year-old woman treated 15 days after symptom onset). Upper row, SLO images: **(A)** preoperative large submacular hemorrhage; **(B)** early postoperative image showing gas tamponade with inferior displacement of the hemorrhage; **(C)** 12-month visit showing absorbed hemorrhage with macular pigmentary changes. Lower row, spectral-domain OCT: **(D)** preoperative hemorrhagic pigment epithelial detachment with subretinal hemorrhage; **(E)** early postoperative scan after hemorrhage displacement; **(F)** 12-month scan showing a restored foveal contour but persistent complete ELM/EZ disruption. Decimal BCVA remained at 0.06 at 12 months.

### 3.4 Anti-VEGF treatment burden and complications

Patients received a mean of 1.8 postoperative anti-VEGF injections (median, 2; IQR, 1.3–2.0) during the 12-month follow-up. No intraoperative retinal tear, vitreous rehemorrhage, or uncontrolled bleeding occurred, and no postoperative endophthalmitis or clinically apparent retinal toxicity attributable to TNK-tPA was observed (0/10; exact 95% CI, 0%–30.8%); given the small sample size, uncommon toxicity cannot be excluded. One 81-year-old phakic patient with pre-existing nuclear lens opacity, who was unable to maintain face-down positioning for physical reasons, showed postoperative cataract progression during follow-up.

### 3.5 Factors associated with 12-month visual outcomes

The 12-month logMAR BCVA was strongly correlated with hemorrhage area (Spearman *r_s_* = 0.87, *P* = 0.001) and time to surgery (*r_s_* = 0.84, *P* = 0.002), and moderately correlated with preoperative CMT (*r_s_* = 0.67, *P* = 0.035); correlations with baseline logMAR BCVA, sex, and age were not statistically significant (all *P* > 0.08). In univariable linear regression, a larger hemorrhage area (*β* = 0.020 per disc area, 95% CI, 0.009–0.032, *P* = 0.004; *R*^2^ = 0.667) and a longer time to surgery (*β* = 0.026 per day, 95% CI, 0.009–0.042, *P* = 0.007; *R*^2^ = 0.624) were associated with worse 12-month logMAR BCVA. Exploratory two-covariate models were highly sensitive to model specification: with baseline logMAR BCVA as the covariate, the coefficient for time to surgery was 0.024 per day (95% CI, 0.007–0.042, *P* = 0.014; adjusted *R*^2^ = 0.560); with hemorrhage area and time to surgery modeled together, the coefficient for hemorrhage area was 0.013 per disc area (95% CI, 0.001–0.025, *P* = 0.038) and that for time to surgery was attenuated (*β* = 0.015 per day, 95% CI, −0.001–0.031, *P* = 0.061; adjusted *R*^2^ = 0.750), reflecting the strong collinearity between these two clinical variables (Spearman *r* = 0.918). Given the small sample size and this collinearity, adjusted estimates are unstable, are reported descriptively with confidence intervals, and should be regarded as hypothesis-generating only; no independent causal effects can be inferred. These analyses are summarized in Table 3. Individual-patient data are presented in Figure 5 (scatter plots of the 12-month logMAR BCVA vs. time to surgery and vs. hemorrhage area) and Figure 6 (line graph of individual BCVA trajectories with anti-VEGF injection timing). Influence diagnostics identified one high-leverage observation (the eye treated 60 days after symptom onset; leverage = 0.95, Cook's distance = 21.7 in the univariable time-to-surgery model). In leave-one-out analyses, the direction and statistical significance of the Spearman associations were retained in every iteration for both time to surgery (*r_s_* range, 0.78–0.90; all *P* ≤ 0.013) and hemorrhage area (*r_s_* range, 0.81–0.93; all *P* ≤ 0.008), indicating that these principal associations were not driven by any single patient, although the magnitude of the linear coefficients varied (time-to-surgery *β* range, 0.024–0.070 per day). The discrepancy between the significant rank correlation and the non-significant univariable linear association for preoperative CMT suggests that any such relationship is not adequately captured by a simple linear model and was therefore interpreted with caution.

**Figure 5:**
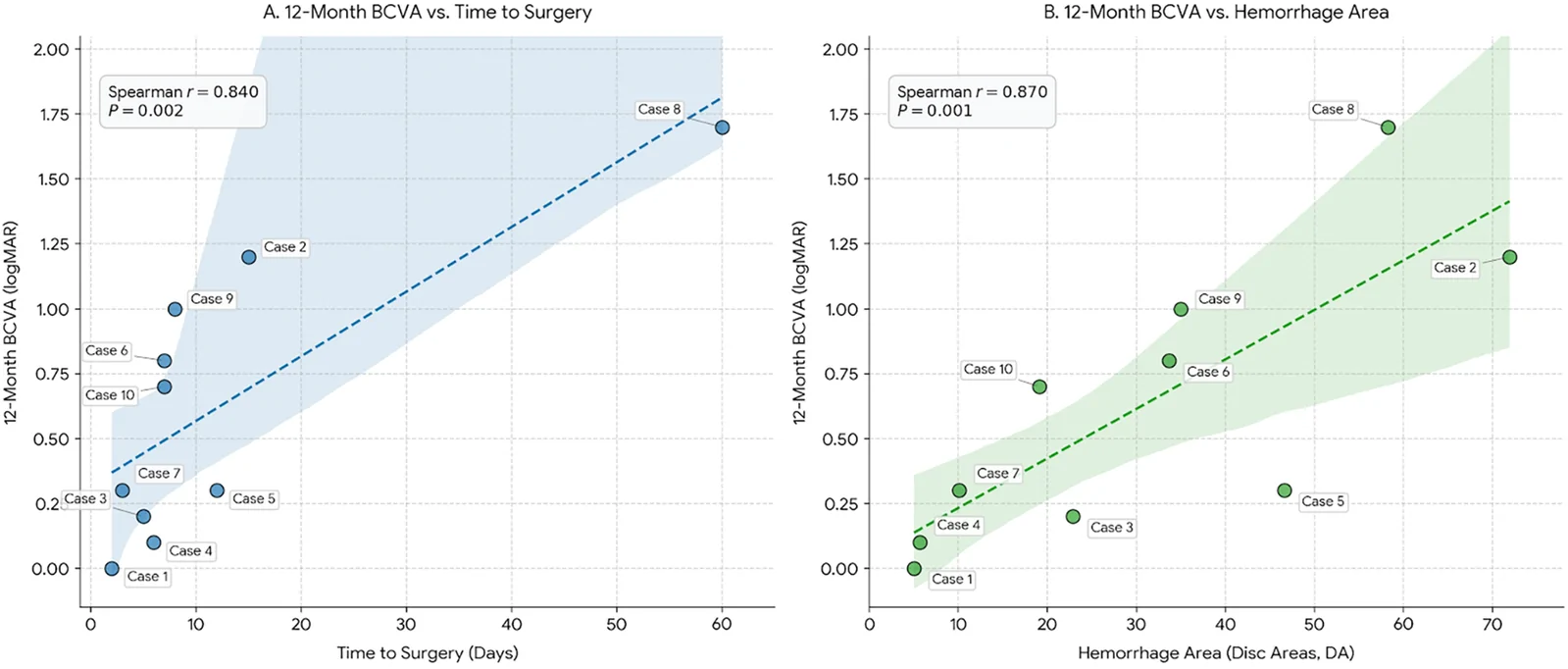
Correlations between 12-month postoperative visual acuity, time to surgery, and submacular hemorrhage area. Scatterplots displaying individual patient data (Case 1–Case 10) for 12-month BCVA in logMAR plotted against **(A)** time to surgery (in days; Spearman *r_s_* = 0.840, *P* = 0.002) and **(B)** baseline submacular hemorrhage area (in disc areas, DA; Spearman *r_s_* = 0.870, *P* = 0.001). Dashed lines represent univariable linear regression trendlines (*β* = 0.026 per day and *β* = 0.020 per DA, respectively), accompanied by surrounding shaded bands indicating the 95% confidence intervals (95% CIs) of the linear fit. Non-parametric Spearman rank correlation coefficients (*r_s_*) and two-tailed *P* values are presented in the top-left insets to reflect monotonic associations, accounting for potential non-normality and high-leverage observations (e.g., Case 8 treated at 60 days). Lower logMAR values correspond to superior visual acuity. Given the strong collinearity between the two predictors (*r_s_* = 0.918) and the small sample size (*n* = 10), these associations are intended as exploratory and hypothesis-generating.

**Figure 6:**
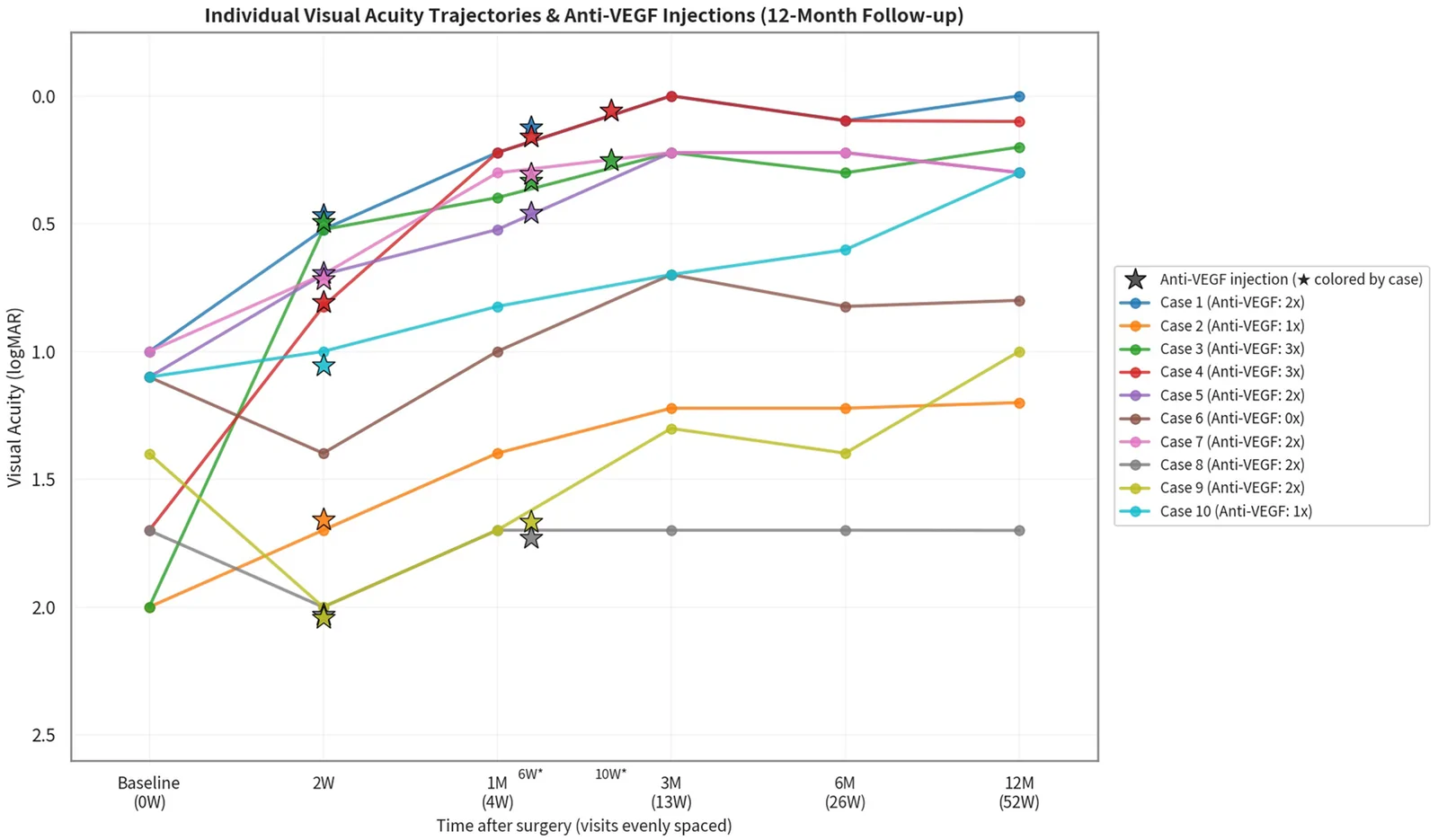
Individual visual acuity trajectories and anti-VEGF injections over 12 months. Line graph illustrating the longitudinal visual acuity (BCVA in logMAR) trajectories for 10 individual cases (Case 1–Case 10) from baseline to the 12-month follow-up visit; scheduled visits are plotted at evenly spaced positions on the *x*-axis. The *y*-axis is inverted such that higher vertical positions represent superior visual acuity. Star markers (★), colored to match the corresponding case trajectory, denote the timing of postoperative intravitreal anti-VEGF injections (at postoperative weeks 2, 6, and 10 according to each eye's retreatment course; injections at weeks 6 and 10 are plotted on the interpolated trajectory between scheduled visits). Overlapping patient trajectories (e.g., Case 8 remaining near 1.7 logMAR throughout follow-up) and concurrent injection markers are rendered with semi-transparency and slight vertical offsets to ensure distinct traceability for each case. The primary endpoint was assessed at 12 months (52 weeks). Case identifiers and the total number of anti-VEGF injections received over 12 months are indicated for each trajectory in the figure key.

## 4 Discussion

In this retrospective case series of PCV complicated by SMH larger than 5 disc areas, vitrectomy with subretinal TNK-tPA injection and gas tamponade achieved complete extrafoveal displacement of the hemorrhage by postoperative day 1 in all eyes. CMT decreased markedly, PED resolved in most eligible eyes by 2 months, and BCVA improved significantly and remained improved at 12 months. No clinically apparent procedure- or drug-related adverse events were observed. To our knowledge, few case series have focused specifically on subretinal TNK-tPA for PCV-related SMH.

Large PCV-related SMH is uncommon but potentially devastating. Its acute and unpredictable presentation makes recruitment of a large, homogeneous single-center cohort difficult. In addition, experimental evidence indicates that subretinal blood can damage photoreceptors rapidly through fibrin-mediated traction and blood-related toxicity (7, 8), creating practical and ethical challenges for delayed-treatment comparisons. Carefully characterized case series can therefore provide useful preliminary evidence for surgical decision-making, although they cannot establish comparative effectiveness.

### 4.1 Rationale for subretinal TNK-tPA

Subretinal administration delivers the thrombolytic agent directly into the hemorrhagic compartment, ensuring immediate contact with the clot. This approach is particularly suited to large or thick SMH because it allows targeted delivery throughout the clot during vitrectomy (12, 13). Although comparative studies have not established the superiority of subretinal over intravitreal administration, both approaches have achieved effective hemorrhage displacement in selected patients (23, 24). Tenecteplase has theoretical advantages over alteplase in this setting, including approximately 14-fold greater fibrin specificity, a longer plasma half-life, and greater resistance to plasminogen activator inhibitor-1 (18, 19). These properties may support sustained clot-directed fibrinolysis after a single administration, although their clinical relevance in the subretinal space remains uncertain. Experimental safety data support further evaluation of a 50 μg ocular dose. In rabbit eyes, intravitreal TNK-tPA at doses up to 50 μg produced no retinal toxicity on clinical examination, electroretinography, or histopathology, whereas higher doses were toxic (28). In a porcine model, subretinal administration of 50 μg TNK-tPA showed no outer retinal toxicity compared with balanced salt solution; RPE defects were confined to the injection sites and were considered procedure-related (29). Preclinical ocular toxicity evaluation of subretinal TNK-tPA has been established primarily up to a single-site dose of 50 μg (29), and direct empirical toxicity data for a single subretinal bolus exceeding 50 μg remain scarce; the only direct clinical report, a single case of PCV-related SMH treated with a subretinal 100 μg (0.1 mL) tenecteplase injection, showed no retinal toxicity, with stable electroretinography and full visual recovery (30). In the present cohort, 7 eyes received a single-site injection of 50 μg, and 3 eyes with extensive multi-quadrant hemorrhage received a cumulative dose of up to 100 μg (0.1 mL) under a spatial dose-fractionation strategy: the dose was distributed across multiple extrafoveal sites, each site received no more than 50 μg (0.05 mL), and the dose delivered within 1 disc diameter of the foveal center was strictly capped at 50 μg, so that the foveal region was never exposed to more than 50 μg and the localized concentration at any single subretinal site remained within the preclinically established 50 μg safety benchmark. We hypothesize that the high fibrin affinity of tenecteplase may allow preferential binding to the dense subretinal fibrin matrix, theoretically limiting free-drug diffusion toward the neurosensory retina, although this mechanism remains speculative. No clinical or OCT signs of drug-related retinal toxicity—such as new pigmentary alterations or progressive outer retinal loss beyond the hemorrhage-affected area—were observed over 12 months of follow-up; nevertheless, evidence for subretinal doses above 50 μg remains anecdotal, and formal dose-escalation toxicity studies are warranted. Limited clinical reports have also been encouraging: intravitreal TNK-tPA combined with anti-VEGF therapy and gas (20) and subretinal TNK-tPA during vitrectomy (21) were associated with visual improvement in AMD-related SMH. In a recent prospective study of vitrectomy with subretinal TNK-tPA and conbercept, 70% of enrolled eyes had PCV and 75% achieved 0.5 logMAR or better at 6 months (22). Our findings in a PCV-only cohort are consistent with these reports.

### 4.2 Comparison with alternative strategies

Pneumatic displacement with or without intravitreal tPA is less invasive than vitrectomy but may be less suitable for thick or longstanding clots and cannot address coexisting vitreous hemorrhage (10, 11). In PCV-related SMH, Kitagawa et al. (17) reported visual improvement after intravitreal tPA, ranibizumab, and gas. Kimura et al. (15) achieved complete hemorrhage displacement in all 15 treated eyes (mean interval from onset to surgery, 9.5 days), with significant visual improvement at the 12-month follow-up. Chen et al. (16) reported improved vision after vitrectomy with subretinal rtPA in 31 patients with PCV-related SMH (mean preoperative duration, 22.6 days), but simultaneous anti-VEGF injection was not associated with additional visual benefit. In the randomized STAR trial, adjusted visual outcomes were comparable between vitrectomy with subretinal tPA and intravitreal tPA with pneumatic displacement (24). The STAR trial was not designed or powered to establish equivalence between the two approaches, and its authors explicitly cautioned against that interpretation. Whether direct subretinal lysis offers any specific advantage for clearing dense subretinal clots—such as those typical of PCV-related hemorrhages, which tend to be larger, thicker, and more exudative—remains an open question. The ongoing pan-European TIGER trial is comparing vitrectomy with subretinal tPA, gas, and aflibercept against aflibercept monotherapy for SMH secondary to exudative AMD (31), but it is not specific to PCV. Previous studies have provided limited information on prognostic factors and subsequent treatment burden. Although our study lacked a control group, vitrectomy with subretinal TNK-tPA injection and gas tamponade was associated with complete early hemorrhage displacement, marked anatomical and visual improvement, a high rate of PED resolution, no clinically apparent TNK-tPA-related toxicity, and an observed mean of 1.8 anti-VEGF injections over 12 months. Earlier intervention and smaller hemorrhage area were also associated with better visual outcomes. Controlled studies are needed to confirm these findings and compare treatment strategies.

### 4.3 Time to surgery and visual prognosis

Time to surgery was strongly correlated with the 12-month visual acuity (Spearman *r* = 0.84, *P* = 0.002). This finding is consistent with experimental evidence of rapid photoreceptor injury after subretinal hemorrhage (7, 8) and clinical reports associating earlier surgery with better visual outcomes (32). Consistent with these findings, a recent study of vitrectomy with subretinal tissue plasminogen activator, pneumatic displacement, and intraoperative anti-VEGF for submacular hemorrhage secondary to PCV or nAMD likewise identified hemorrhage duration as the most critical prognostic factor, with significantly better visual outcomes in eyes treated within 14 days (33). Hemorrhage area showed the strongest univariable association with 12-month acuity (Spearman *r* = 0.87, *P* = 0.001). In exploratory two-covariate models, the adjusted coefficients were sensitive to model specification: the coefficient for time to surgery was 0.024 per day (*P* = 0.014) when baseline logMAR BCVA was the covariate, but was attenuated (*P* = 0.061) when hemorrhage area was also included, reflecting the strong collinearity between these two variables (*r* = 0.918); these adjusted estimates should therefore be interpreted descriptively rather than as evidence of independent effects. Eyes with a preoperative duration exceeding ten days already showed outer retinal disruption on OCT, whereas promptly treated eyes preserved the outer retinal bands and achieved better acuity, in one case reaching 1.0 decimal vision. These exploratory findings highlight the importance of prompt assessment and suggest that early surgical intervention may be considered when a large, thick submacular clot is present.

### 4.4 Sustained outcomes and anti-VEGF treatment burden over 12 months

A key strength of the current study is its minimum 12-month follow-up duration with standardized longitudinal evaluation. Our 12-month data demonstrate that the rapid visual recovery achieved after subretinal TNK-tPA injection is not merely transient but sustained over a full year, with median logMAR BCVA remaining improved at 0.30. Disease control was achieved with a mean of 1.8 injections (median 2, IQR, 1.3–2.0) over the 12-month follow-up. Because this was a single-arm study, we report the injection burden descriptively and make no comparative claim of reduced treatment burden. For context, the monthly or bimonthly regimens used in the EVEREST (34), EVEREST II (35), and PLANET (36) trials, and anti-VEGF monotherapy under standard proactive regimens (such as Treat-and-Extend) or clinical trial protocols typically entailing 7 to 11 injections in the first year to maintain visual and anatomical gains (37), illustrate the treatment intensity usually needed for active PCV; these figures are provided only as context, because differences in study populations (none of these trials enrolled eyes with large submacular hemorrhage), PED characteristics, and treatment protocols preclude direct comparison of injection requirements. Notably, PCV lesion activity tends to diminish after a large subretinal hemorrhage—an effect associated with rupture of major polyps—with a reported median injection-free period of 12 months after hemorrhage (25). This natural history likely contributed to the low observed injection requirement in our cohort. Pharmacologic monotherapy also appears insufficiently potent against PED: in a 2-year conbercept study (mean 8.6 ± 5.4 injections), complete macular dryness was achieved in 88% of eyes, yet PED persisted in 24%, with serous PED-associated polyps requiring a higher treatment load (median 11 vs. 3.5 injections) (38). This clinical challenge is further underscored by the 6-year PLANET extension data, in which 69.4% of eyes still had polypoidal lesions at year 2 and patients received a mean of 10.2 ± 2.9 additional anti-VEGF injections between years 2 and 6 (39).

Compared with the incomplete PED resolution reported in some studies of anti-VEGF monotherapy, the high rate of complete PED resolution observed in our series (90.0% by month 2) is clinically noteworthy. However, because clot evacuation, gas tamponade, and anti-VEGF therapy were applied in combination, this observation cannot be attributed to any single component, and spontaneous quiescence of the polypoidal complex after large hemorrhage (25) offers an alternative explanation. Whether subretinal TNK-tPA directly promotes PED resolution—for example, by altering the sub-RPE environment—remains a hypothesis that requires experimental confirmation. Moreover, this study lacked a control group and did not directly evaluate the underlying polypoidal lesions, so the contribution of subretinal TNK-tPA and the mechanism of PED resolution remain uncertain.

### 4.5 Limitations

This study has several limitations. Its retrospective, single-arm design and small sample size preclude causal inference and comparative effectiveness claims. One eye with an exceptionally long treatment delay (60 days) exerted high leverage in the regression models; although leave-one-out analyses confirmed the robustness of the principal rank associations, the precision of the effect estimates remains limited. The correlation and regression analyses were exploratory and further constrained by the strong collinearity between hemorrhage area and intervention timing; therefore, adjusted estimates should not be interpreted as definitive independent causal effects. Requiring a minimum 12-month follow-up may introduce selection and attrition bias: 11 eyes underwent the surgical procedure, but 1 eye was excluded because its follow-up was shorter than 12 months, and its outcomes are not captured in this report; complete follow-up among the 10 analyzed eyes does not eliminate this potential source of bias, although consecutive enrollment of all eligible eyes partly mitigates this concern. The modest cohort reflects the low clinical incidence of large (>5 DA) PCV-related SMH presenting within an actionable surgical window at a single tertiary center. The absence of a control group (e.g., intravitreal TNK-tPA or anti-VEGF alone) means that the incremental benefit of subretinal delivery remains to be quantified. While the treatment regimen is supported by prior preclinical and clinical evidence and showed favorable safety without observed toxicity over 12 months, the small sample size limits the statistical power to rule out uncommon adverse events. Despite these limitations, the complete displacement of subfoveal hemorrhage in all eyes by postoperative day 1, the high rate of complete PED resolution (90.0% by month 2), the standardized 12-month follow-up demonstrating sustained visual improvement, and the coherent association between time to surgery and visual prognosis strengthen the clinical relevance of our findings. Future prospective, multicenter registry studies with larger cohorts are warranted to validate these preliminary findings and further investigate the mechanisms underlying polyp regression and PED resolution.

Taken together, the consistent and rapid anatomical response, the absence of observed drug-related toxicity, and the coherent association between time to surgery and visual outcomes provide a rationale for future controlled studies.

## 5 Conclusion

In this small retrospective series, vitrectomy with subretinal TNK-tPA injection and gas tamponade, followed by individualized anti-VEGF therapy, was associated with rapid hemorrhage displacement, PED resolution, improved macular anatomy, and significant visual improvement through 12 months, with an observed mean of 1.8 anti-VEGF injections over 12 months and no observed drug-related adverse events. Earlier surgery and smaller hemorrhage area were associated with better visual outcomes. These findings are preliminary and require confirmation in prospective controlled studies, and the mechanisms underlying polyp stabilization and PED resolution after this technique remain to be clarified.

## Data Availability

The original contributions presented in the study are included in the article/supplementary material, further inquiries can be directed to the corresponding author/s.

## Supplementary material

SUPPLEMENTARY VIDEO S1 The subretinal tenecteplase (TNK-tPA) injection procedure.
